# Relationship between tendon structure, stiffness, gait patterns and patient reported outcomes during the early stages of recovery after an Achilles tendon rupture

**DOI:** 10.1038/s41598-020-77691-x

**Published:** 2020-11-27

**Authors:** Didier Laurent, Lorcan Walsh, Amir Muaremi, Nicolau Beckmann, Eckhard Weber, Frederique Chaperon, Harry Haber, Joerg Goldhahn, Andrea Sabine Klauser, Michael Blauth, Matthias Schieker

**Affiliations:** 1 Translational Medicine Department, Novartis Institute for BioMedical Research, Fabrikstrasse 10-3.40.4, 4002 Basel, Switzerland; 2 Novartis Ireland Ltd., Elm Park, Merrion Road, Dublin 4, Ireland; 3 Musculoskeletal Diseases Area, Novartis Institutes for Biomedical Research, 4002 Basel, Switzerland; 4 Institute for Translational Medicine, ETH Zürich, HCP H 15.3, Leopold-Ruzicka-Weg 4, 8093 Zürich, Switzerland; 5 Department of Trauma Surgery, Center Operative Medicine, University Hospital, Anichstrasse 35, 6020 Innsbruck, Austria; 6 Department of Radiology, Medical University Innsbruck, Anichstrasse 35, 6020 Innsbruck, Austria

**Keywords:** Physiology, Biomarkers, Medical research, Signs and symptoms

## Abstract

After an Achilles tendon (AT) injury, the decision to return to full weightbearing for the practice of sports or strenuous activities is based on clinical features only. In this study, tendon stiffness and foot plantar pressure, as objective quantitative measures that could potentially inform clinical decision making, were repeatedly measured in 15 patients until 3 months after the AT rupture by using shear wave elastography (SWE) and wearable insoles, respectively. Meanwhile, patient reported outcomes assessing the impact on physical activity were evaluated using the Achilles Tendon Total Rupture Score (ATRS). At week-2 post-injury, stiffness of the injured tendon varied from 6.00 ± 1.62 m/s (mean ± SD) close to the rupture to 8.91 ± 2.29 m/s when measured more distally. While near complete recovery was observed in distal and middle regions at week-8, the shear wave velocity in the proximal region recovered to only 65% of the contralateral value at week-12. In a parallel pre-clinical study, the tendon stiffness measured in vivo by SWE in a rat model was found to be strongly correlated with ex vivo values of the Young’s modulus, which attests to the adequacy of SWE for these measures. The insole derived assessment of the plantar pressure distribution during walking showed slight sub-optimal function of the affected foot at week-12, while the ATRS score recovered to a level of 59 ± 16. Significant correlations found between tendon stiffness, insole variables and distinct ATRS activities, suggest clinical relevance of tendon stiffness and foot plantar pressure measurements. These results illustrate how an alteration of the AT structure can impact daily activities of affected patients and show how digital biomarkers can track recovery in function over time.

## Introduction

Achilles tendon (AT) tears happen mostly in recreational athletes (75%)^1^ and over 80 percent of AT ruptures also occur during sport or recreational activities^2^, causing substantial morbidity, impairment of mobility, and loss of work time^1,3^. With the aging population becoming more active, the incidence of AT ruptures has been increasing over the past decade^4–6^, for examples in middle-age patients from 1.8/10,000 to 2.9/10,000 between 2003 and 2013 in the province of Ontario, Canada^5^ and reaching in Sweden 5.5/10,000 and 1.47/10,000 for men and women, respectively^4^. Tendon injuries are often treated with a variety of non-surgical (e.g., immobilisation, ice, physical therapy) and surgical interventions, along with reduction of physical activities to allow for healing of the tendon^7,8^.

Tendons are mostly made of highly structured collagen fibers (60% of dry mass) that connect and transmit forces from muscle to bone^9^. They are able to store elastic energy and withstand the high tensile forces upon which locomotion is entirely dependent. Optimal tendon stiffness is critical for an effective muscle–tendon interaction. The course of recovery of normal tendon stiffness after injury is poorly understood, making it difficult to objectively determine when the tendon has healed sufficiently and has the functional capacity to allow the patient to return to normal activities or sports.

Conventional imaging modalities such as ultrasound (US) and magnetic resonance imaging (MRI) can monitor changes in tendon morphology over time. Yet, these imaging modalities do not provide an objective assessment of tendon healing based on recovery of its biomechanical characteristics. A technique that allows for reliable non-invasive assessment of tendon mechanical properties in vivo may have significant clinical impact. This tool should enable for the monitoring of tendon changes as a result of injury, pathology, and/or treatment and support decision making to return to sports activities.

US-based shear wave elastography (SWE) is an innovative technique that quantitatively assesses tissue stiffness^10^. Local tissue strain is produced using acoustic radiation force by focused US impulses that induce the formation of shear waves. The shear wave velocity is measured using high-frequency US imaging from which tissue stiffness is then inferred^11^. Under well controlled conditions^12^, this technology appears sensitive enough to detect significant differences in the AT stiffness during extension, in neutral position and during maximum dorsiflexion^13^. Greater levels of stiffness have been shown in those physically active compared to non-active subjects^14^.

Achilles tendons exhibit strong anisotropy, which translates to a greater stiffness when placing the US transducer parallel, as opposed to perpendicular, to the tendon fiber orientation^15^. Overall, stiffness along the Achilles tendon may exceed 800 kPa, the upper limit of measurement for the most advanced SWE device available on the market. However, stiffness values in tendons with full-thickness rupture can vary from ~ 3 to ~ 220 kPa, depending on the healing stage^16^. Due to the rupture, it is possible that the tendon stiffness is heterogeneous, reflecting a loss of parallel arrangement of collagen fibers in the injured area. Collagen abnormalities in healing tendons may result in less resistance to tensile forces, and it is therefore important to carefully monitor stiffness changes along the tendon length during recovery.

Achilles tendon injuries impair the ability to walk normally, and surgical or therapeutic interventions aim to restore a healthy gait cycle. Recent advances in wearable technologies for gait analysis include the development of inertial wireless sensors, optical motion trackers, portable force plates, insole pressure sensors and wireless electromyography^17^. Sensorised wearable plantar pressure insoles have been used to capture dynamic stability during single- and dual-walking tests in clinical settings^18^ and were proposed to capture real-world data over multiple days^19^. Wearable insoles offer an objective insight into weight-bearing through each foot during walking and offers a means to capture foot kinematics, thus providing the potential to measure functional changes during rehabilitation.

The Achilles Tendon Total Rupture Score (ATRS) is an established and validated patient-reported outcome score used for clinical assessment^20^, however it only captures patient self-reported function. The ATRS seems reliable for the comparison of groups of patients but, with a minimal detectable change of 18.5 in a scale of 100, may have only limited use for the repeated assessment of individual patients in the clinic^21^.

As a premise to preclinical/clinical translation and future research, a validation study aimed at comparing tendon stiffness measurements by SWE with ex vivo measurements of the Young’s modulus was performed in a rat model of tenotomy. Once the validity of this measure was demonstrated, the main purpose of the present human proof-of-concept study was to explore, from a comprehensive approach relying on the use of relatively new technologies, the relationship between tendon stiffness measured by SWE, load distribution in the foot sole during walking measured by sensorised insoles and patient-reported outcomes.

## Results

Of the eighteen patients who participated in the study, fifteen patients completed the study up to 12 weeks and three patients discontinued the study prior to week-2 measurements, two of them due to patient decision and one due to a post-procedural complication. For all patients, the rupture occurred in the middle portion of the tendon. In addition, 9 out of 18 patients were surgically treated while the remaining ones received a conservative treatment for the AT rupture, keeping the ankle from moving by using a walking boot with heel wedges. During the treatment period, the walking boot was removed to allow for all the evaluations reported here; however, it was used again readily after the assessments.

### Tendon structure

**Figure 1:**
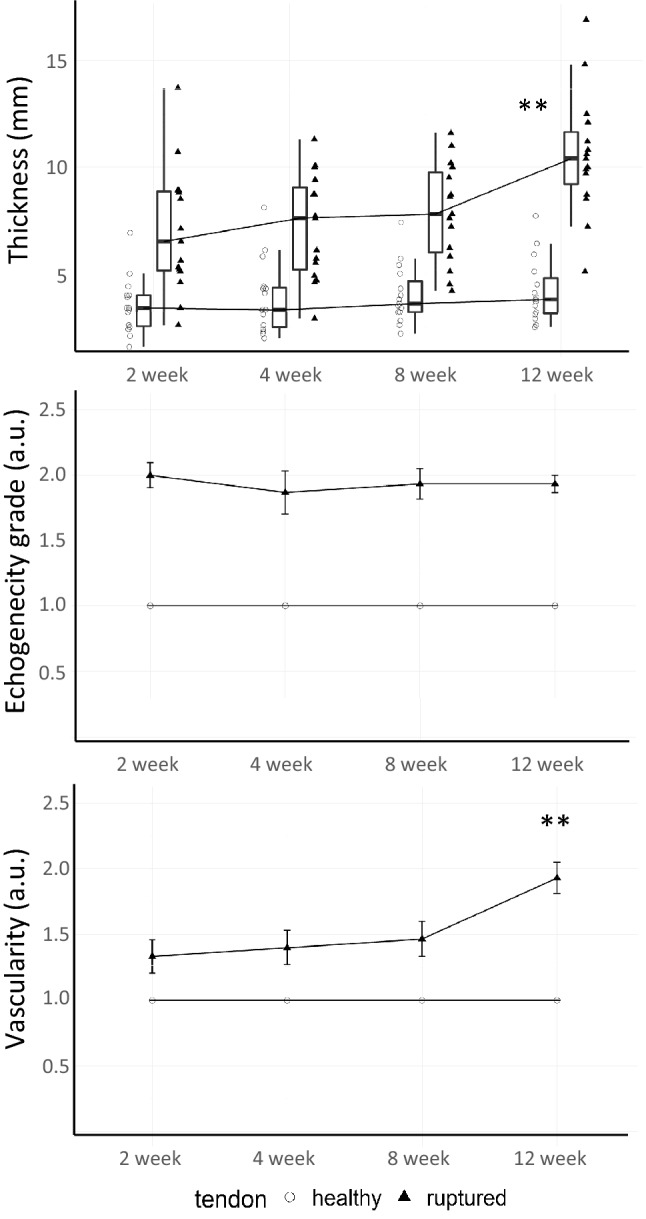
Mean structural changes observed in the ruptured region of the injured tendon throughout the 12-week monitoring period. Thickness and echogenicity measured by B-mode US and vascularity assessed by Power Doppler. Values (N = 15/timepoint) are medians ± SD for the boxplot and means ± s.e.m. for the two line plots. Both echogenicity and vascularity are expressed in arbitrary units (a.u.). **p < .0001 vs baseline in injured leg (repeated measure ANOVA).

Unlike in the contralateral tendon, tendon thickening, high echogenicity and Power Doppler US derived patterns of vascularity were observed in the injured tendon throughout the 12-week observation period (Fig. 1). Interestingly, while the increased vascularity was already present at 2 weeks post injury, it kept increasing until week 12 along with thickening of the tendon. Ultimately, the injured tendon became ~ threefold thicker than the contralateral healthy tendon at the end of the observation period.

### Tendon stiffness

**Figure 2:**
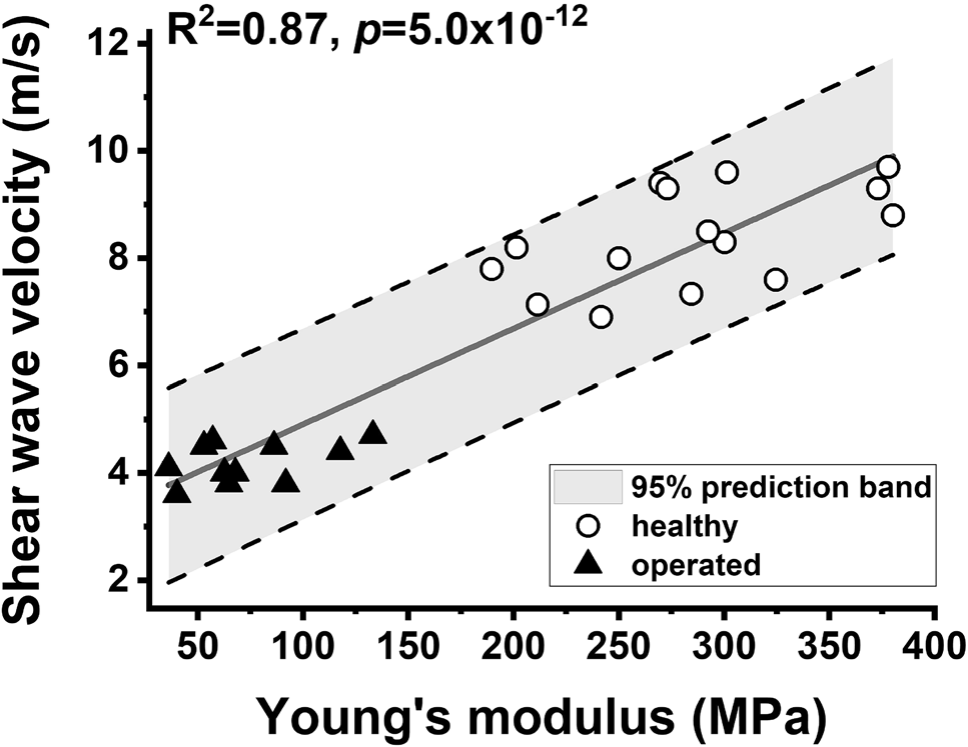
Comparisons between shear wave velocities in rat Achilles tendons in vivo and post-mortem analyses of biomechanical properties of the same tendons. Measurements were performed at 4 weeks after tenotomy and on a separate group of healthy rats. Significant correlations were obtained between shear wave velocity and the Young’s modulus (R^2^ = 0.87, p = 5.0 × 10^–12^, as indicated) and the maximum tensile stress (R^2^ = 0.81, p = 9.0 × 10^–10^, data not shown). The significant correlations between in vivo and ex vivo data provided a valid basis for clinical assessments of injured tendons by SWE.

Pre-clinical rat model: A strong correlation was found between tendon stiffness measured in the pre-clinical model of tendon injury by SWE and ex vivo measures of the Young’s modulus (Fig. 2), which provides a valid basis for clinical assessments.

**Figure 3:**
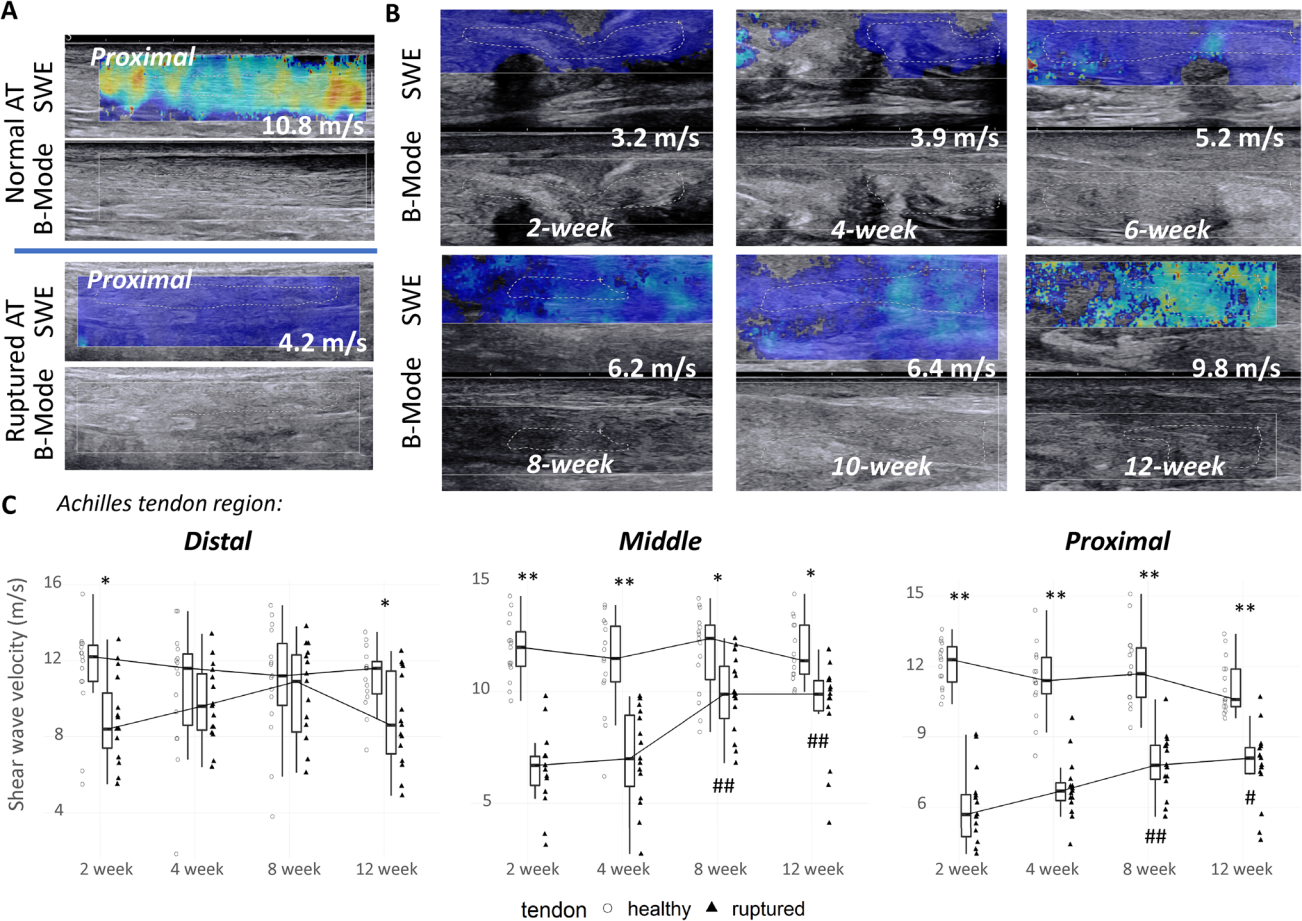
**(A)** Examples of tendon stiffness maps and corresponding B-mode images from the proximal region of injured and contralateral healthy AT of a patient two weeks after the rupture. SWE color maps are coded from blue to red, indicating low to high tissue stiffness. Note the low shear wave velocity values (ie, low stiffness) measured in the injured Achilles tendon. **(B)** SWE maps from a patient AT indicating a progressive increase in shear wave velocity over the 3-month observation period after the rupture. **(C)** Average time-course recovery of the injured AT showing approximately 65% recovery in tendon stiffness until 12 weeks after the injury. Values in the boxplots (N = 15/timepoint) are medians ± 1.5*Interquartile Range (IQR). *p < .05, **p < .0001 vs injured tendon; ^#^p < .05, ^##^p < .0001 vs baseline in the injured tendon.

Clinical assessments: SWE maps recorded from the damaged tendon of a patient at different time-points during the healing period are shown in Fig. 3A,B and corresponding changes in tendon stiffness measured from the entire group of patients are displayed in Fig. 3C. Tendon stiffness in contralateral healthy tendons appeared homogeneous and stable over the 12-week observation period (shear wave velocity mean ± SD at baseline in distal: 11.43 ± 2.48 m/s, middle: 11.90 ± 1.35 m/s and proximal: 12.09 ± 0.95 m/s regions of the tendon). It should be noted that the evaluation of SWE measurement variability from test/retest of healthy tendons at week 2 and week 4, a sufficiently short period of time for no change to occur in the contralateral tendon, showed a CV% in distal, middle and proximal regions of 11.6%, 11.2% and 11.0%, respectively, which attests to the good reproducibility of SWE measurements. When measured close to the zone of rupture (middle-proximal) and shortly after the rupture (week 2), shear wave velocity values were lower in injured tendons (middle: 6.53 ± 1.71 m/s, proximal: 6.00 ± 1.62 m/s) in comparison with healthy tendons. However, it was reduced to a lesser extent (8.91 ± 2.29 m/s) when measured distally from the ruptured area (Fig. 3C). Near complete recovery of tendon stiffness was observed in distal and middle regions at approximately eight weeks post-injury. Indeed, the shear wave velocity reported from the distal and middle regions of ruptured tendons was 10.30 ± 2.36 10–11 m/s and 9.87 ± 1.70 m/s, respectively, which is close or only slightly lower than the velocity measured in corresponding regions of the contralateral tendon (distal: 10.87 ± 2.97 m/s, middle: 11.79 ± 1.84 m/s). On the contrary, in the proximal region, shear wave velocity recovered only to about 65% (7.77 ± 1.60 m/s) of the value measured in the healthy tendon at week 12 after the injury (Fig. 3C).

### Foot gait pressure and patient reported outcomes

**Figure 4:**
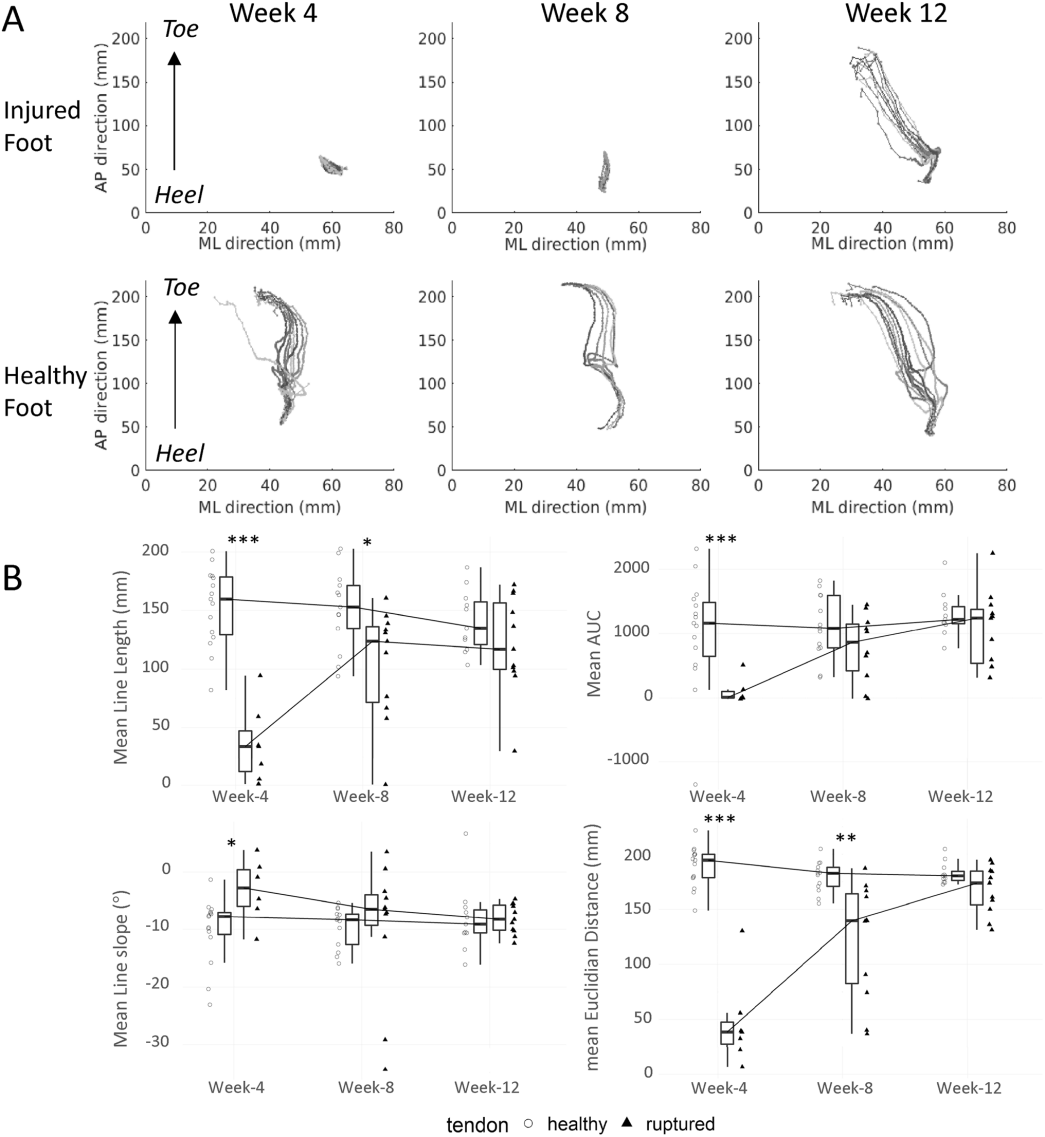
**(A)** Example of weight pressure dynamic assessments recorded by the digital insole system from a patient at week 4, 8 and 12 during the healing period. Each dotted line represents Centre-of-Pressure (CoP) path data recorded from one of the few steps managed during the walking exercise (5 to 10 steps in average). Data obtained from the injured foot are shown on the top row and those obtained from the healthy foot on the bottom row. **(B)** Time-course changes in CoP variables over the 12-week observation period. Values in the boxplots (N = 15, 13 and 11 at week 4, 8 and 12 respectively) are medians ± 1.5*Interquartile Range (IQR). *p < .05, **p < .01, ***p < .001 vs injured tendon.

Gait pattern analysis (Insole data): Plantar centre of pressure (COP) data collected throughout the healing period from a typical patient using the Pedar insole system is presented in Fig. 4A. This patient recovered to almost normal gait patterns during the 3-month rehabilitation shown by use of the entire surface of the foot across multiple steps, in particular placing pressure further towards the toes increasingly over subsequent weeks. At week 4, on the injured side, the patient applied weight through the heel region only, while after 3 months of recovery, the patient could distribute weight from the heel towards the toes as well as across the medio-lateral axis, in accordance with what can be observed on the normal, contralateral leg. Yet, the proportion of time during which this patient placed pressure through the toes in the injured leg remained diminished compared to the healthy leg.

At the patient group level, changes in the COP variables during recovery are summarized in Fig. 4B. As shown for the COP line length, COP area under the curve (AUC), line slope and Euclidean distance, the plantar pressure profile of the injured foot returned towards normal values at the end of the observation period. At this stage, patients began to apply pressure through their toes. However, for 3 out of the 4 variables displayed, all potential indicators of the smoothness in how the foot moves through the gait cycle, the body weight transfer on the foot sole still appeared incomplete at week-12, possibly indicating that torsional properties of the injured foot were not fully re-established at this time of the recovery.

**Figure 5:**
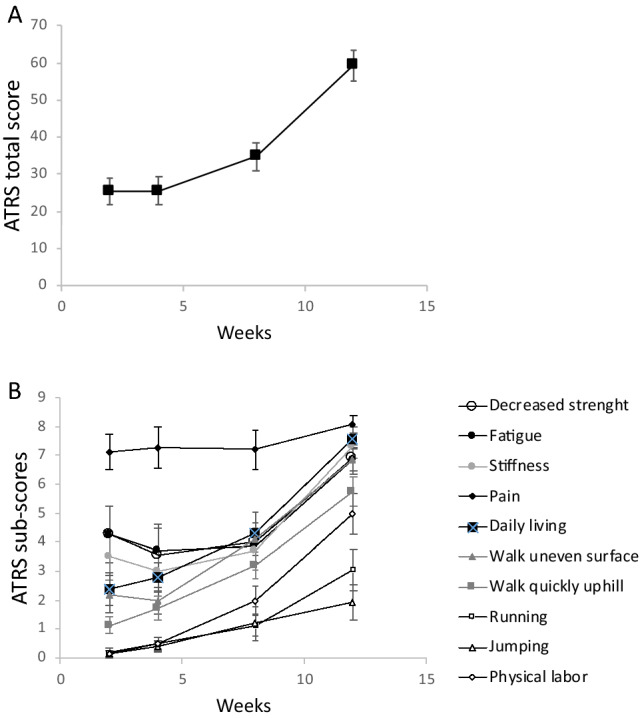
Time-course recovery of the Achilles Tendon Total Rupture Score (ATRS) score **(A)** and related distinct activities **(B)** over the 12-week observation period after the AT rupture. Note that pain did not appear to be a major cause of reduction in the total ATRS, however patients still remained limited at week 12 in the practice of strenuous activities such as running, jumping, and walking on uneven surfaces. Note also that activities for most of them pertain to the perception of what patients thought they could do, not the activity level actually managed in the last few days prior to the assessment. Values (N = 15/timepoint) are means ± s.e.m.

PROM (ATRS score): Full weight-bearing capacity was reached in the injured leg at week 12 post-injury, with a steady improvement over the 3-month observation period. However, based on ATRS results, patients were limited by symptoms during the first 8 weeks post-injury and were able to recover ~ 60% of their perceived functionality at week 12 (Fig. 5A). Looking further into ATRS activities (Fig. 5B), it appears that patients were still not confident in their ability to jump, run, do physical labor, and walk fast and uphill. This is different from other activities like walking on uneven surfaces and activities of daily living, as well as symptoms like whole-body stiffness, low strength and fatigue, which all showed some improvement from week 2 to week 8 and marked improvement between week 8 and week 12. Interestingly, while patients were on standard-of-care pain medication, pain remained unchanged throughout the recovery period and hence was deemed as being not a major cause of reduction in the total ATRS score.

### Correlations between weight-bearing, tendon stiffness and patient reported outcomes

Significant correlations were found between weight-bearing variables extracted from the insole measurements and the AT stiffness from the affected foot as well as selected ATRS distinct activities performed over the 3-month observation period (Table 1). These positive correlations illustrate the role of the tendon in the distribution of weight at the level of the foot (in particular on the heel-toe axis) and highlight the importance of the functionality in the realization of activities requiring force, speed and good balance across both legs.

**Table 1:** Correlations between changes in foot plantar pressure variables as derived from the Center of Path (CoP, the weight distribution pathway at the foot arch during a step), selected ATRS activities and tendon stiffness over the 12-week recovery.

		In-shoe insole variables					
		CoP-Length	CoP-Slope	CoP-AUC	CoP-Euclidian Length	AP distance	ML distance
Tendon stiffness (% healthy)		0.49 (p = .028)	− 0.19 (p = .434)	0.44 (p = .067)	0.44 (p = .055)	0.28 (p = .139)	0.18 (p = .357)
ATRS score		0.50 (p = .025)	− 0.02 (p = .929)	0.65 (p = 0.004)	0.74 (p < 0.0001)	0.51 (p = 0.004)	0.67 (p < 0.001)
ATRS strenuous activities	Walking on uneven surface	0.62 (p = .003)	0.041 (p = .866)	0.70 (p = .001)	0.82 (p < .0001)	0.77 (p < .0001)	0.60 (p < .001)
	Running	0.38 (p = .099)	− 0.07 (p = .787)	0.63 (p = .005)	0.61 (p = .004)	0.57 (p = .001)	0.42 (p = .024)
	Jumping	0.29 (p = .220)	− 0.10 (p = .674)	0.53 (p = .023)	0.47 (p = .034)	0.42 (p = .022)	0.40 (p = .033)

Note that ATRS activities are used as indicators of the perception of what patient think they can do, not as indicators of what patients actually do at the evaluation time.

## Discussion

With this longitudinal study, we investigated the recovery of tendon structure, biomechanics, gait patterns and patient reported outcomes over 12 weeks after AT rupture. We analyzed how reliable and sensitive ultrasound-based SWE could be in detecting changes in AT stiffness during healing, and studied correlation of these respective changes with standard US features (i.e., tendon thickness, echogenicity and macro-vascularization), foot plantar pressure changes and patient reported outcomes (ATRS).

Distinct softening of the tendon has previously been detected by using SWE in Achilles tendinopathy^22^, with SWE showing excellent sensitivity, specificity and accuracy when compared with clinical examination. Furthermore, a strong correlation was found for SWE with conventional US findings^22,23^ as well as with histological assessments^24^ and the apparent elastic moduli of human cadaveric Achilles tendon given by tensile tests^25^. Finally, our own preclinical studies in rats establish SWE as a tool for assessing changes in the biomechanical properties of the AT following rupture. The mechanism driving the biomechanical change observed during healing after a tendon rupture likely stems from changes in the collagen structure of the tendon^26^ which could affect fluid flow through the ECM^27^ and cross links between collagen molecules^28^. Our study demonstrated that SWE can be considered as a reliable and sufficiently sensitive approach for the detection of a defective area within the tendon itself as well as to monitor the time-course recovery of tendon stiffness after a rupture. While stiffness appeared fairly homogeneous throughout the healthy tendon, spatial differences have been detected along the length of the injured tendon. Thus, when measured in the longitudinal plane, the shear wave velocity in the injured tendon was greater and closer to normal in areas away from the rupture zone. On the contrary, for measurements performed close to the rupture zone and in the relaxed state (i.e., foot at 90° position), shear wave velocity was as low as ~ 50% of the normal value within ~ 2 weeks after the injury. This emphasizes the importance of a good localization of the measurements to collect precise data, and for this the use of tissue landmarks such as the distance from the insertion point on the calcaneus is proposed.

The second most striking result of this study was that, even though weight-bearing capacity for most patients was almost fully recovered after three months, shear wave velocity remained ~ 40% lower in the injured (proximal) area compared to the healthy tendon, which itself remained stable over the 12-week observation period. On this last point, it is interesting to note that other studies have reported a decrease in stiffness in the non-ruptured side^29,30^. While recent SWE data also showed that repaired tendons gradually become stiffer postoperatively^31^, our results clearly indicated that tendon healing is still incomplete within 3-month after the rupture, even though patients at this stage typically reached a range of motion from the ankle to the foot that is at least sufficient enough for short distance normal walking. In fact, the incomplete tendon healing suggested by our SWE data is consistent with a recent study showing that the healed AT after rupture has ~ 50% lower stiffness even after a ~ 6-year healing phase^32^. This lack of total recovery of the AT stiffness may help to understand why patients at the end of the 3-month observation period still seemed limited in their perceived ability to perform strenuous activities that create high-strain events for the AT such as jumping, running, fast and uphill walking, as revealed by results of the ATRS symptoms. In spite of a near absence of pain during the recovery period, after 12 weeks of recovery, there may still be a risk of re-rupture while performing activities associated with strong elongation of the tendon. The measurement of tendon stiffness by SWE could be particularly useful to identify the risks associated with the practice of high-impact sport shortly after an AT injury. We show in this study that the ATRS proved to be highly reactive and clinically relevant since it has demonstrated patient recovery up to a score of ~ 60 (out of 100 at most) at 12 weeks, a value slightly higher than that of other studies^33^, but which could testify to the effectiveness of the recovery protocol used in this study.

Healing of an injured tendon results from different processes which can overlap in their duration depending on the location and severity of the injury. The initial inflammatory stage not only involves infiltration of cells such as neutrophils, monocytes and macrophages but also secreted angiogenic factors that initiate the formation of a vascular network^9^. Such neo-vascularization may be responsible for the survival of the newly forming fibrous tissue at the injury site^34^. The tendon thickening, higher Archambault echogenicity grade and Power Doppler US patterns of vascularity observed here would indeed suggest the presence of swelling in the ruptured area throughout the three-month healing time. During the repair process, recruited fibroblasts may also contribute to the synthesis of various components of the extracellular matrix^35^ leading to the absorption of large amounts of water in the injured tendon. To which extent this explains the thickened tendon observed in the last month of the monitoring period remains to be verified. However, the fact that tendon stiffness was still lower than normal suggests that, at this stage, collagen fibers were probably not yet organized optimally for exercise at high strain magnitude.

Correlations found here between tendon stiffness and foot plantar pressure results attest to the role of the AT in the transmission of force necessary to generate toe-off during the late stance of the gait cycle. This can therefore lead to the assumption that only when the AT stiffness approaches normal values, gait patterns return to normal. The foot regains the ability to distribute load adequately on the entire surface of the sole allowing the patient to improve his/her ability in performing strenuous exercises. Such an assumption can in fact be supported by the recent data showing that a stiff AT reduces ground contact time during drop jumps^36^. Furthermore, recent studies have shown that not only the structure of the tendon, but also its stiffness in the first 12 weeks of recovery could be linked to gait symmetry at 24 weeks, which may indicate the prognostic value of these measures for long-term outcome after AT rupture^37^. Interestingly, we also found that the higher the tendon stiffness, the lower the standard deviation associated with the measurement of the CoP-Length was during the walking test made of ~ 10 steps (r = − 0.48, p < 0.05). This lower variability in how plantar pressure was distributed during the step may be related to the recovery of some balance between the two feet when walking, although further studies are needed for a better understanding of this. Other COP-derived variables were extracted from these data, including the COP line slope and mean mediolateral distance, however significant associations were not found against SWE or ATRS activities for all of these variables.

Our study has limitations. First, both its heterogeneity, due to the presence of patients with and without surgery, and its limited sample size make difficult to show strong correlations between each of the variables measured independently, and hence permits for proof of concept only. However, this study was realized at a single site, thus allowing to minimize some of the variability inherent to the techniques used (e.g., operator-dependent ultrasound measurements). Second, with regard to foot plantar pressure assessments, we only assessed short walking distances. The results of the walking test would benefit from a greater number of steps for a more sensitive detection of impairment in specific gait characteristics. While this may significantly increase the amount of data collected, the automations developed as part of this work should however allow for a rapid extraction and analysis of specific gait variables. Third, while the ATRS questionnaire was mainly developed to provide an index of patient performance, subcategories were used here to provide information on the patient’s ability to perform particular exercises that were more or less strenuous for the AT. The validity of these sub-category tests taken separately will have to be studied further, before drawing definite conclusions on their association with foot pressure variables. Lastly, our patient cohort is clinically inhomogeneous. However, the objective of the study was not to precisely assess the speed of healing after an ATR. We studied the correlation between tendon biomechanics, the dynamics of weight loading on the foot and patient symptoms. The incorporation of patients from a broader spectrum of disease allowed to establish such correlations.

In conclusion, we could show correlations of tendon structure, biomechanics, gait patterns and patient reported outcomes over 12 weeks during recovery after AT rupture. We could demonstrate that SWE is an accurate diagnostic tool that can improve detection of tendon injuries and might thus be well suited for monitoring treatment effects aiming to accelerate the regeneration of injured tendon. In addition, this study has shown the link between structural and biomechanical characteristics of the Achilles tendon with foot plantar pressure and patient’s perception of being able to perform a certain type of exercise, thus allowing a better understanding of how an alteration of the AT structure can have an impact on the daily activities of affected patients.

## Material and methods

### Patient population

The study population comprised 18 male and female patients (18 years and older) with confirmed acute unilateral AT total or partial rupture requiring orthopedic treatment which consisted of either a conservative or a minimally invasive surgical treatment procedure. Patients with partial rupture were selected based on sudden pain onset, no positive results to calf squeeze or Matles tests and absence of partial tears due to tendinosis, verified by ultrasound. Patients with AT tendinopathy without partial rupture (as verified by ultrasound) and sudden pain onset were excluded from the study. Although, patients with total ATR and patients with partial rupture should not be combined to investigate the recovery rate after injury, we consider these groups relevant to investigate the relationship between AT stiffness, gait pattern and patient symptoms, which are all a priori adequate measures for each of these conditions.

Due to the exploratory nature of the study, no formal sample size estimation could be done. Yet, the sample size of 18 patients was deemed practical and feasible based on prior studies^11^ and assumed to allow detection of changes that are clinically relevant to future intervention studies. Exclusion criteria were: Evidence of systemic acute or chronic inflammatory disease other than at the AT, previous history of ipsilateral AT injuries, pre-existing ipsilateral tendinopathy and conditions that prevented increasing weight-bearing during rehabilitation, such as lower limb injuries, cognitive impairment, or other conditions impacting controlled increase in weight-bearing were all considered as exclusion criteria. Of the 18 patients who participated in the study, 15 patients completed the study. Two patients discontinued the study due to personal reasons not related to the study and one due a post-surgical complication. Patient demographics and baseline characteristics are presented in Table 2.

**Table 2:** Patients demographics and baseline characteristics for the 15 patients that completed the study.

		Type of tendon rupture		
		Total N = 12	Partial N = 3	Overall N = 15
Age (years)	Mean (SD)	49.7 (12.50)	52.7 (5.03)	50.3 (11.31)
Sex—n (%)	Male	11 (92%)	3 (100%)	14 (89%)
	Female	1 (8%)	0	1 (11%)
Height (cm)	Mean (SD)	177.4 (5.64)	177.0 (3.61)	177.3 (5.15)
Weight (kg)	Mean (SD)	84.0 (12.81)	91.2 (7.42)	85.6 (12.00)
BMI (kg/m^2^)	Mean (SD)	26.7 (3.99)	29.1 (2.63)	27.2 (3.79)

The treatment was allocated to each patient per standard of care at the Center Operative Medicine of the Innsbruck University Hospital and is described in Table 3. For 14 of the 15 patients who completed the study, treatment was initiated in the first week after the injury, with the exception of one patient (patient 1001–1007) with partial rupture whose conservative treatment started on day 9, and all patients wore a removable boot for up to 10 weeks. From the beginning, weight-bearing was allowed, and all patients were encouraged to walk after subsidence of initial pain with a modified shoe allowing for rolling the foot despite restricted dorsiflexion of the foot. After removal of the boot, passive and active range of motion as well as physiotherapy with increasing load was performed on each patient according to an individualized program. Experimental procedures were approved and carried out in accordance with the guidelines of the human investigation committee of the Innsbruck School of Medicine. All subjects gave informed consent after the purpose, nature, and potential risks of the study were explained to them.

**Table 3:** Treatment allocated to each patient.

Patient #	Affected side	Exact primary diagnosis	Time of treatment start post-ATR	Time of surgery post-ATR	Cast/boot period
1001–1001	Right	A	–	Day 6	Drop out: no further information available
1001–1002	Right	A	–	Day 7	8 weeks
1001–1003	Left	A	Day 2	–	8 weeks
1001–1004	Left	A	Day 1	–	10 weeks
1001–1005	Right	A	Day 1	–	8 weeks
1001–1006	Left	A	Day 2	Day 3	6 weeks
1001–1007	Left	B	Day 9	–	8 weeks
1001–1009	Right	A	Unknown	Day 2	7 weeks
1001–1010	Left	A	Day 1	–	8 weeks*
1001–1011	Right	B	Day 1	–	6 weeks
1001–1012	Right	A	Day 1	–	8 weeks
1001–1013	Right	A	Day 1	Day 1	3.5 weeks
1001–1014	Left	A	Unknown	Day 2	4 weeks
1001–1015	Left	A	Day 1	Day 2	7 weeks
1001–1016	Left	B	Day 1		8 weeks
1001–1017	Right	B	Unknown	Day 5	5.5 weeks
1002–1001	Unknown	A	Unknown	Day 2	Drop out after V3
1002–1002	Unknown	A	Unknown	–	Consent withdrawn after V1

*ATR* Achilles tendon rupture (occurred on Day 1).

A: Acute total ATR without symptoms of tendinopathy before.

B: Partial rupture with sudden pain but no positive calf squeeze test or Maties test, absence of tears verified with ultrasound.

*The boot was extended for 4 weeks for this patient, but only when he was carrying heavy loads.

### Study design

A maximum of 25-day screening period was allowed for each patient. Eligible patients underwent first evaluations 2 weeks after surgery or, for patients treated conservatively, 2 weeks after confirmed diagnosis of tendon rupture. Tendon stiffness, thickness and vascularity of both legs were evaluated by ultrasound (US) imaging using shear wave elastography (SWE), B-mode and Power Doppler, respectively. To study the correlation between tendon stiffness and foot motion and clinical improvement, weight-bearing was assessed, and patients were asked to complete the ATRS, a patient reported outcome measurement (PROM) measure to evaluate their symptoms and the impact on physical activity. Patients were followed throughout the course of tendon healing over three months. Assessments were performed at the hospital at weeks 2, 4, 8 and 12 between January 2017 and July 2017.

### Ultrasound measurements

AT rupture damages the organized architecture of the collagen fibers at the rupture site. During the healing process, the AT undergoes remodeling which leads to changes in the structural and biomechanical properties as well as vascularity of the tendon. We therefore assessed tendon stiffness, thickness, echogenicity and vascularity by US imaging using a dedicated sonography unit (Aixplorer, Supersonic Imagine; Aix-en-Provence, France; software version 5). Both the affected and unaffected (contralateral) tendon were evaluated during the healing period at weeks 2, 4, 8 and 12, with the same settings applied in every subjects. Week-2 was the first possible measurement time for all patients, and for patients that were treated conservatively, the walking boot was removed to allow for the various assessments. Tendon stiffness was measured using the SWE mode on the Aixplorer, while B-mode and Doppler ultrasound acquisitions were performed for structural (ie, thickness and echogenicity) and vascularity assessments, respectively. All measurements were done in triplicates, in the sagittal plane, with the subject lying in the prone position with both arms alongside and both feet hanging over the edge of the stretcher and the foot under a relaxed/neutral position, hence the plantar flexors were in the passive state. The measurement session started only after a 10-min period of rest on the examination bed to equilibrate fluids in the body.

For all measurements, the high-resolution linear 15 MHz transducer (SuperLinear SL15-4, SuperSonic Imagine) was stationed as lightly as possible on top of a generous amount of coupling gel, perpendicularly to the surface of the skin. The transducer was angled cranially-caudally until the scanned plane showed an AT with maximum echogenicity. Thickness, echogenicity, vascularity and shear wave velocity were all measured in the same regions of each AT. Three representative locations in the proximal, middle and distal end of the tendon were considered for the analysis. For SWE measurements, the transducer was kept motionless for 3 to 5 s during the map acquisition. Finally, slow flow settings and color mode were selected for Power Doppler measurements in small vessels.

Specifically for the SWE system, all measurements were performed using the musculoskeletal mode with a frequency range of 4–15 MHz, the SWE Opt as the penetration mode, the opacity at 85% and presets adjusted to a depth of 2 cm and an elastic scale from 0 to 800 kPa. The color scale used in the shear modulus (in kPa) showed the lowest values in blue to the highest values in red. The size of the regions of interest (ROI) had to be at least 40 ∗ 15 mm in order to cover one third of the Achilles tendon and the Q-Box diameter was defined by the thickness of the tendon, which is the distance between the superior and inferior borders of the Achilles tendon^38^. The Q-Box was traced manually to include maximum of the tendon third and to avoid peritendinous structures like the paratenon.

During measurements, sufficient ultrasound gel was applied between the skin and the transducer to avoid skin deformation. The midpoint of the transducer was placed perpendicularly on the skin’s surface on every tendon third with a light pressure, and then the SWE mode was activated to examine the shear wave modulus of the tendon^39^. During the SWE acquisition, the transducer was kept motionless for about 3–5 s until the gray scale image showed the tendon in its longitudinal section. Image quality was closely monitored throughout the measurements. As soon as the color appeared uniform in the selected tendon region, with superior and inferior borders of the tendon continuously visible, images were frozen, then put on the Q-Box to depict a shear wave modulus map and get it stored for SWE analysis (kPa, m/s)^40^. Three images were captured at each measurement site of each tendon third. The mean of the elastic modulus from all three images was used for further analyses.

For image analysis, regions-of-interest (ROI) were selected so that their diameter covered almost the entire thickness of the tendon at a given location. SWE color maps were analyzed quantitatively for tendon stiffness ROI-based determination of shear wave velocities (in m/s) calculated from triplicate measurements and assuming that the shear wave velocity can be an index for quantifying Young’s modulus of the tendon^41,42^. Tendon thickness was measured from B-mode ultrasound images. Regarding the longitudinal grading of tendon echotexture based on echogenicity, the grading system was applied as follows^43^: Grade 1—normal-appearing tendon with homogeneous fibrillar echotexture and parallel margins; Grade 2—focal fusiform or diffuse enlarged tendon with bowed margins; Grade 3—hypoechoic areas (ruptures) with or without tendon enlargement, accompanied by signs of fiber dehiscence (ie, rupture gap). Finally, AT vascularity was evaluated using Power Doppler. Although the vascularization in normal AT is very poor, after a tendon total rupture, healing was shown to be accompanied by neovascularization and increased vascularity^34^. Therefore, video clips were graded at each tendon location as Grade 0 for no intra-tendinous vascularity, Grade 1 for 1/3 intra-tendinous vascularity, Grade 2 for 2/3 intra-tendinous vascularity and Grade 3 for 3/3 intra-tendinous hypervascularity.

For validation purposes, a pre-clinical experiment was conducted on both healthy and tenotomized rats using the same imaging equipment adopted for the clinical assessments and a 25 MHz transducer (SL25-15, SuperSonic Imagine). Studies performed on Sprague–Dawley rats were approved by the Cantonal Veterinary Authorities of Basel, Switzerland (license BS-2439) and performed in accordance with the Swiss animal welfare regulations. Upon onset of anesthesia with isoflurane (Abbott, Cham, Switzerland), the right leg was shaved, and the exposed skin prepared aseptically. A dorsal incision was made above the AT and the superficial tendon was exposed and transected at the mid-portion from the lateral aspect perpendicular to the collagen fibers. Tendon ends were then sutured together using a three loop Pulley pattern. SWE was applied to anesthetized animals in a similar way as described for humans to measure AT stiffness in vivo. Measurements were performed at 4 weeks after tenotomy. Immediately after SWE assessments, rats were culled, and tendons extracted for ex vivo biomechanical assessments using an Instron testing apparatus (model 3300, Instron, Norwood, MA). After mounting the tendons between clamps, stress–strain curves were generated, with elongation applied in the axial direction until rupture.

### Wearable insoles

In the clinical trial, instrumented insoles consisting of a pressure sensitive grid were used to record the average amount of weight applied on the entire foot and to also collect more granular spatio-temporal data on plantar pressure for both the affected and non-affected legs, over a walking test at each visit during the recovery period. These data were recorded using the Pedar in-shoe system (Novel GmbH, Munich, Germany) which has been shown to be a sensitive tool for the assessment of in-shoe plantar pressure distribution^44^. The insole system measured vertical pressure using a matrix of 99 capacitive sensors with a spatial resolution of approximately 10 mm and a working dynamic range of 0–600 kPa at a rate of either 50 or 100 Hz. Each patient was asked to walk in a straight line over 10 m at a self-selected speed during each visit to the site during rehabilitation while data was collected from insoles worn in each shoe. Data were analyzed post-hoc using Matlab (Mathworks Inc., Natick, MA, USA).

**Figure 6:**
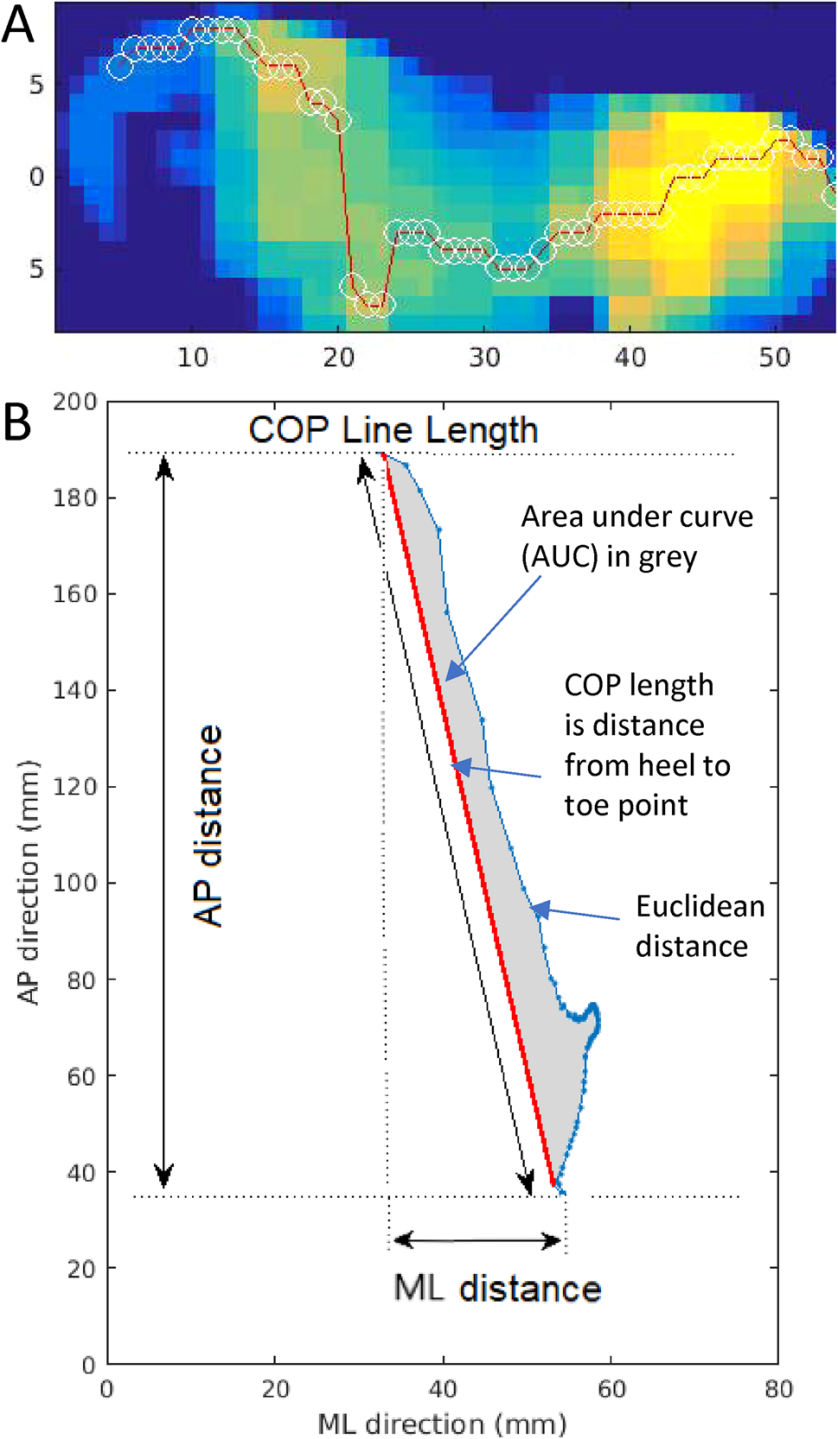
Example of the plantar pressure distribution recorded by the insole system at the beginning of the gait cycle **(A)**. The pressure sensing grid indicates a high load on the heel side (yellow) and a low load on the toe side (blue). White circles through the red line show the average point through which pressure is applied, while the red line illustrates the path followed by center-of-pressure (CoP) based measures over one step. Corresponding profile of CoP data from the same step for this patient, including few extracted features such as the medial–lateral (ML) and anterior–posterior (AP) distances, the CoP line length in blue, the area under the curve in grey and the CoP length in red **(B)**.

As recently described^45^ and shown in Fig. 6, a frame of pressure data (${f}_{x,y}^{i}$ ), measured in kPa, was collected for each time instant (i) for all sensors in the mediolateral (x) and anteroposterior (y) directions. The sum of this force across all sensors for each time instant i, ${F}_{i}={\sum }_{x=1}^{Nx}\sum_{y=1}^{Ny}{f}_{x,y}^{i}$, where Nx and Ny are maximum number of sensors in the x and y direction, was manually examined post data collection to identify the start and end of walking periods. Sensor failure resulted in intermittent empty data frames and these were removed from further analyses. Data were further manually reviewed to identify where numerous empty data frames significantly affected analyses and the corresponding steps were not included in any analysis. An empirically defined threshold applied to the force data was used to define the start and end of each step. The centre of pressure (COP_x,y_^i^) in the x and y directions at each time instant i for each step was extracted for further analysis as per the following equations:

$${COP}_{x,y}^{i} = [{COP}_{x}^{i}, {COP}_{y}^{i} ]$$

$${COP}_{x}^{i}= \frac{{\sum }_{x=1}^{Nx}\sum_{y=1}^{Ny}{POS}_{x} \times {f}_{x,y}^{i}}{{F}_{i}}$$

$${COP}_{y}^{i}= \frac{{\sum }_{x=1}^{Nx}\sum_{y=1}^{Ny}{POS}_{y} \times {f}_{x,y}^{i}}{{F}_{i}}$$

where ${POS}_{x}$ and ${POS}_{y}$ are the positions of each sensor (from 1 to m and n respectively) in centimeters along directions x and y, ${COP}_{x}^{i}$ and ${COP}_{y}^{i}$ are the centres of pressure along respective directions at time i.

The COP for a single step during one of the walks is shown in Fig. 6. The point through which force was applied over the entire step is shown starting from heel strike (occurring low in the AP direction) to toe off (occurring high in the AP direction). The following metrics were extracted from these data:

- AP distance (mm): The difference between the minimum and the maximum of COP across the antero-posterior (y) direction was calculated for each step. The overall mean of this distance was recorded across all steps per walk.
- COP line length (mm): The COP line was defined as a straight line between the COP point at heel strike and at toe off. The length of this line was defined as the COP line length.
- COP-path length (mm): The sum of the Euclidean distances between each successive COP point (the COP-path).
- COP-AUC (mm^2^): The area under the curve between the COP-path and the COP line.
- Weight-bearing: Over each walk, the maximum force placed on both foot (the weight-bearing capacity) was recorded.

### Achilles tendon rupture score (ATRS)

The Achilles Tendon Total Rupture Score (ATRS) was developed to evaluate patient-reported symptoms and their effects on physical activity following either conservative or surgical treatment of an AT rupture^20^. The score consists of ten items evaluating clinically relevant aspects of symptoms and physical activity. Each item ranges between 0 (no limitation) and 10 (severe limitation) on a Likert scale with a maximal score of 100 indicating no symptoms and full function. Patients completed the ATRS at screening and at weeks 2, 4, 8 and 12.

### Statistical analysis

All statistical analyses were performed using SAS, Version 9.4. Descriptive statistical methods were used to calculate the means ± standard deviations (SD) of tendon shear wave velocity, thickness, echogenicity and vascularity grades, ATRS scores and insoles-derived variables. Test–retest reliability of the tendon stiffness measurements across all visits used data from healthy contralateral tendons and the coefficient of variation (%CV) was calculated for the proximal, middle and distal tendon region. No imputation was done for missing values. A repeated measures analysis of variance (ANOVA) was done with fixed effects of region of index, time, and foot position. The difference of SWE values between the damaged and contralateral tendon was examined using paired Student t-test. A cutoff value between injured and healthy tendons was evaluated by receiver operating characteristic analysis, choosing a confidence interval of 95%. The correlation between quantitative SWE values, clinical scores and insoles variables was evaluated using Spearman rank correlation coefficients. All reported p-values are two sided, and p-values < 0.05 were considered to be statistically significant.

## Abbreviations

AT: Achilles tendon
ATRS: Achilles total rupture score
CoP: Center-of-pressure
PROM: Patient reported outcome measurement
SWE: Shear wave elastography
US: Ultrasound
